# The dynamics of social cohesion in response to simulated intergroup conflict in banded mongooses

**DOI:** 10.1002/ece3.8475

**Published:** 2021-12-20

**Authors:** Elizabeth F. R. Preston, Faye J. Thompson, Solomon Kyabulima, Darren P. Croft, Michael A. Cant

**Affiliations:** ^1^ Centre for Ecology and Conservation College of Life and Environmental Sciences University of Exeter Exeter UK; ^2^ Banded Mongoose Research Project Queen Elizabeth National Park Uganda; ^3^ Centre for Research in Animal Behaviour College of Life and Environmental Sciences University of Exeter Exeter UK; ^4^ Institute for Advanced Study Berlin Germany

**Keywords:** aggression, cooperative breeding, fighting, grooming, Intergroup conflict, post‐conflict behavior, social cohesion, warfare

## Abstract

Intergroup conflict is widespread in nature and is proposed to have strong impacts on the evolution of social behavior. The conflict–cohesion hypothesis predicts that exposure to intergroup conflict should lead to increased social cohesion to improve group success or resilience in future conflicts. There is evidence to support this prediction from studies of affiliative responses to outgroup threats in some animal societies. However, most of these studies have focused on behavioral changes over short time periods (minutes and hours after exposure to an outgroup), and hence very little is known about the dynamics and durability of responses to intergroup conflict over the longer term. We investigated this question by simulating intergroup encounters in wild banded mongooses (*Mungos mungo*) and measuring social behavior before, during, and after these encounters over a 5‐day period. We also ran control trials with non‐threatening stimuli. Banded mongooses reacted immediately to intrusion stimuli by vocalizing, grouping together, and advancing on the stimulus. In the first 5 min after simulated intrusions, we saw an elevation in grooming levels, but in the hour after exposure grooming rates declined sharply, contrary to our expectation. In the two subsequent days, grooming rates remained at this depressed rate. In control trials, the initial increase in grooming was not seen, but grooming declined compared to the longer‐term time periods. Grooming changed across time, but not in the same pattern as during intrusions, suggesting that intrusions had an impact above and beyond that of the experimental setup. The dynamics of grooming responses were short lived and more complex than we initially expected. We suggest this unexpected result may be linked to the frequency of aggressive intergroup encounters in this system. As control and experimental trials were run at different times of year, future work would be needed to confirm that these relative patterns are replicable. Our results indicate short‐lived impacts of outgroup threat on measures of social cohesion in this species, but cannot confirm longer‐term changes.

## Graphical abstract

The conflict–cohesion hypothesis predicts that exposure to intergroup conflict should lead to increased social cohesion to improve group success or resilience in future conflicts. We investigated this question by simulating intergroup encounters, and control trials, in wild banded mongooses (*Mungos mungo*) and measuring social behavior before, during, and after these encounters over a 5‐day period. Our results indicate short‐lived impacts of outgroup threat on measures of social cohesion in this species; longer‐term impacts could not be confirmed.

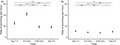

## 1 INTRODUCTION

Conflict between groups over scarce resources, often referred to as intergroup conflict, can have a strong influence on fitness costs and benefits of social behavior (Rusch & Gavrilets, 2016; Thompson, Marshall, Vitikainen, & Cant, 2017; Van Belle et al., 2014). Costs arise as a consequence of mortality or injury (Manson & Wrangham, 1991; Plowes & Adams, 2005; Rosenbaum et al., 2016; Thompson, Marshall, Vitikainen, & Cant, 2017), loss of resources, or energetic costs of fleeing. Conflicts can also bring individual and group benefits, for example, through increased access to resources or mating opportunities (Arseneau et al., 2015; Cant et al., 2002; Harris, 2010), or via group augmentation or group winner effects, because larger groups are often more successful during intergroup fights, and can therefore acquire or defend valuable resources or territories (Cassidy et al., 2015; Cheney & Seyfarth, 1987; Clutton‐Brock, Gaynor, et al., 1999; Clutton‐Brock, O’Riain, et al., 1999; Gros‐Louis et al., 2003; Markham et al., 2012; Sillero‐Zubiri & Macdonald, 1998). Theoretical models developed to understand the evolution of human cooperation suggest that intergroup conflict can influence the evolution of within‐group social traits (Bowles, 2006; Henrich, 2004). Specifically, in these models, the propensity to attack other groups coevolves with a propensity for altruism within human groups because the individual fitness costs of expressing an altruistic trait are outweighed by the group (or kin group) fitness benefits that are enjoyed by groups with many altruists (Bowles, 2006; Choi & Bowles, 2007; Henrich, 2004; Lehmann, 2011; Rusch & Gavrilets, 2016). Mortality rates from intergroup conflicts in chimpanzee (*Pan troglodytes*) societies and banded mongooses (*Mungos mungo*) (Johnstone et al., 2020) are comparable to those of subsistence human hunter‐gatherer and farmer societies (Wrangham et al., 2006 – although there is controversy surrounding human mortality estimates), suggesting that the theoretical models developed to explain human cooperation could apply to some non‐human animals, especially those with similar social systems, ecology, and type of intergroup encounters (Cant et al., 2016; Wrangham et al., 2006), and to banded mongooses in particular as modeled by Dyble, 2021. High mortality rates might be necessary for long‐term behavioral responses to intergroup conflict to evolve due to the high fitness costs associated with these conflicts. In any case, animal models and studies across species are needed to explore this interesting phenomenon.

While these theoretical models are concerned with the coevolution of altruism and intergroup hostility over many generations, empirical studies of intergroup conflict in both human and animal societies have focused on the short‐term behavioral consequences of outgroup threats (see Table 1). One widely held idea is that groups exposed to intergroup conflict should pull together and become more cohesive or affiliative, sometimes called the “conflict‐cohesion” hypothesis (Thompson et al., 2020). In an evolutionary context, this predicted response could be adaptive if increased cohesion or affiliation helps individuals and groups to reduce the costs or realize the benefits of intergroup competition and provide the building block to altruism within groups. The impact of simulated conflict on social cohesion has been tested experimentally in animal societies with mixed results. In green wood hoopoes (*Phoeniculus purpureus*) (Radford, 2008a, 2008b, 2011), dwarf mongooses (*Helogale parvula*) (Morris‐Drake et al., 2019) and cichlid fish (*Neolamprologus pulcher*) (Bruintjes et al., 2015) within‐group affiliation increased after simulated encounters with other groups. By contrast, in capuchin monkeys (*Cebus capucinus*) (Polizzi di Sorrentino et al., 2012), simulated intergroup encounters led to an increase in within‐group aggression, suggested by the authors to indicate high tensions within the group causing deteriorating within‐group relationships. Observational studies have also found contrasting evidence of the effect of intergroup conflict on within‐group behavior, either increasing grooming or increasing aggression (Cooper et al., 2004; Cords, 2002; Payne et al., 2003). With the notable exception of studies of primate ranging behavior (Markham et al., 2012), previous studies have examined only the short‐term impacts of intergroup conflict on within‐group social behavior (i.e., in the minutes and hours after an interaction; Table 1). Although a recent study on chimpanzees has detected changes in intragroup behavior on the day of intergroup encounters, compared to those when it does not happen, and pushes into longer‐term changes showing increased group modularity (how close individuals are in proximity) in months with greater numbers of border patrols or intergroup encounters (however, modularity was not compared to null permutation models, so this result is hard to interpret as group structure may have changed through time randomly and this result is not linked solely to intergroup encounters, Samuni et al. (2020). It is unknown whether intergroup conflict has longer‐term impacts on collective behavior (i.e., that are detectable days or weeks after an intergroup encounter).

**TABLE 1:** Studies assessing the impact of intergroup encounters on social behavior after an intergroup encounter (studies that only measure behavior during an intergroup encounter are not included here as we are focusing on responses after the conflict has ended), and the timescale on which responses were measured

Species	Reference	Observation or Experiment?	Captive or wild?	Time‐point behavior was recorded	Impact on social behavior	Timescale
Wied's black tufted‐ear marmosets, *Callithrix kuhli*	Schaffner and French (1997)	Exp	C	D	**+SM +DB**	10 or 20 min
				B, A	**+Aff +SM**	5 or 10 min
Tufted capuchin monkeys, *Cebus paella*	Polizzi di Sorrentino et al. (2012)	Exp	C	A	**+Agg +DB +DH** (Aff)	10 min
Cichlid fish, *Neolamprologus pulcher*	Bruintjes et al. (2015)	Exp	C	B, A	**+Aff** (Agg)	10 min
				D	**+DB**	10 min
Green wood hoopoe, *Phoeniculus purpureus*	Radford (2008)	Exp	W	B, A	**+Aff**	1 h
Dwarf mongooses, *Helogale parvula*	Morris‐Drake et al. (2019)	Exp	W	A	**+Vig +NN**	1 h
				A	**+Aff** (Agg)	Until 50% start foraging
Mountain gorillas, *Gorilla beringei* *beringei*	Mirville et al. (2020)	Obs	W	B, A	**+Aff (Females, short conflicts)** ‐Agg (Adult male)*	1 h
Samango monkeys, *Cercopithecus mitis erythrarchus*	Payne et al. (2003)	Obs	W	A	**+Aff**	10 min
Bonnet macaques, *Macaca radiate*	Cooper et al. (2004)	Obs	W	B, D, A	**+Agg**	Variable
Ring‐tailed lemurs, *Lemur catta*	Nunn and Deaner (2004)	Obs	Semi‐free‐ranging	B, A	(Agg, Aff)	30 min
				D	**+DB**	Length of encounter
Javan gibbons, *Hylobates moloch*	Yi et al. (2020)	Obs	W	A	**‐Aff**	1 h
Green wood hoopoe, *Phoeniculus purpureus*	Radford (2008)	Obs	W	B, A	**+Aff**	1 h
Green wood hoopoe, *Phoeniculus purpureus*	Radford and Fawcett (2014)	Obs	W	B, A	**+Aff**	1 day
Blue monkeys, *Cercopithecus mitis*	Cords (2002)	Obs	W	A	+Aff**	Unknown

Note

Time‐point behavior was coded as B = before the presentation of stimuli/presence of rival group; D = during the presentation of stimuli/presence of rival group; and A = after the presentation of stimuli/presence of rival group. When behaviors were compared between before and after a (simulated) encounter, it was coded as B, A; whereas when behavior was only recorded afterwards, it was coded as A. When behaviors were only recorded afterwards they were usually compared to controls. Social behaviors were coded as follows: Aff = affiliation including grooming or allo‐preening; Agg = aggression between individuals within the focal group; DB = defensive behaviors including aggression toward the intruders; SM = scent marking; DI = dominance interactions; Vig = vigilance behavior; NN = nearest‐neighbor distance. + and bold typeface indicates an increase in the behavior; − and bold typeface indicates a decrease in the behavior; behaviors in brackets and regular typeface are those which were studied but not affected by intergroup exposure. *This indicates that these results are limited to specific categories of individual and conflict, rather than the whole group, as indicated by the information in brackets ** This indicates an anecdotal record of grooming increase rather than empirical data.

Here, we test the hypothesis that intergroup conflict has lasting impacts on within‐group behavior using simulated intergroup encounters in wild banded mongooses. Banded mongooses are small (<2 kg) diurnal herpestids that live in stable multi‐male, multi‐female groups of between 10 and 30 individuals. They are cooperative breeders, and spend time babysitting, escorting young, and engaging in affiliative ingroup behavior through grooming and scent marking. Dispersal is relatively rare for both sexes and occurs as a result of aggressive eviction (Cant et al., 2013, 2016; Thompson et al., 2016; Thompson, Marshall, Vitikainen, Young, et al., 2017). They differ from other social mongoose species (including meerkats and dwarf mongooses) in their breeding systems, as multiple females give birth synchronously in each breeding attempt (compared to single dominant females in other mongoose species), and offspring are reared cooperatively by the whole group as in other social mongooses (Hodge et al., 2011). Banded mongooses are ideal for this study because groups are highly territorial (defending resources, offspring, and mating opportunities) and engage in frequent aggressive interactions, with substantial costs to adults and offspring (Johnstone et al., 2020; Thompson, Marshall, Vitikainen, & Cant, 2017). We have observed no tolerant or neutral interactions with other groups in over 24 years of study, and encounters seem to be more violent and frequent than those seen in meerkats (Jordan et al., 2007) and dwarf mongooses (Christensen et al., 2016). Intergroup encounters occur mainly between groups when they encounter each other, as groups travel together while foraging, they appear to be reactive in nature, but there is a possibility that some are proactive as females may lead the group into border areas while in estrus (Johnstone et al., 2020). They differ from many primate species, including chimpanzees and humans, in this sense, which often patrol borders actively (Langergraber et al., 2017; Watts & Mitani, 2000).

Following previous studies in primates and other social vertebrates, we use grooming and aggression as measures of group social cohesion (Table 1). We predict that simulated encounters will lead to increased grooming (affiliative behavior) and reduced within‐group aggression (agonistic behavior). We also measure scent marking and alarm calling, two other potentially affiliative collective behaviors, which we predict will increase after simulated intrusions. Unlike studies that compare behavioral responses to intruder stimuli versus controls, on the day of presentations, in this experiment, we measured within‐group social behavior before, during, and in the days after simulated intrusions. Examining the temporal dynamics of social cohesion in this way can help to understand how much behavior changes as a direct result of intergroup conflict, and for how long.

## 2 METHODS

### 2.1 Study site

Data for this study were collected from wild banded mongooses on the Mweya Peninsula in Queen Elizabeth National Park, Uganda (0°12′S, 29°54′E), between March 2016 and May 2017. This banded mongoose population is part of a long‐term study, and detailed descriptions of the study site can be found in Cant (2000); Cant et al. (2013); and Rood (1975). The mongooses in this study are habituated and can be observed closely from 2 to 4 m away with no disturbance to normal behavior. Individuals are marked using individual unique hair‐shave patterns on their backs to allow individual identification. Groups were located using radiotelemetry as one or two mongooses in each group wear radiocollars (which weigh 26–30 g; Sirtrack Ltd, Havelock North, New Zealand) with a whip antenna (20 cm; Biotrack Ltd, Dorset, UK). Five focal groups were used in this study (group size ranged from 7 to 30, counting only individuals over 6 months old), in total 100 individual mongooses were included in the study.

Groups are territorial and defend their territories from other groups during frequent, highly aggressive intergroup conflicts (mean encounter rate per group = 0.8 per week (non‐estrus periods) to 2.9 per week (group estrus); data from 12 groups) (Cant et al., 2002; Nichols et al., 2015; Thompson, Marshall, Vitikainen, & Cant, 2017)). Individuals respond to sighting a rival group by standing alert and giving a specific screeching call known as a “war cry” (Cant et al., 2016), after which group members congregate and stand alert. Groups often approach each other in tight formation, screeching, growling, and feinting toward the opposing group. These face‐offs periodically erupt into chases and fights involving biting and scratching, and sometimes individuals are held down and attacked by multiple rival group members. Intergroup fights can result in serious injury and sometimes death (Cant et al., 2002; Nichols et al., 2015; Thompson, Marshall, Vitikainen, & Cant, 2017). Almost all of these deaths are males (Johnstone et al., 2020). Several lines of evidence suggest that females lead their groups into intergroup encounters in search of extra‐group matings, and use the cover of battle to escape mate guards in their own group (Johnstone et al., 2020).

### 2.2 Experimental design

A single trial of the experiment took place over 5 days. For each intrusion trial in each group, we recorded behavioral data for 2 h per day (1 hour in the morning, and 1 hour in the afternoon at roughly the same times on each day) on Days 1 and 2. This acted as a reference or “before” period to assess changes in behavior in response to a simulated territorial intrusion. On Day 3, we presented two types of stimulus: (1) scents and calls, presented in the morning; and (2) live intruders, presented a few hours later. The rationale for this sequential presentation was to simulate how the mongooses would encounter natural cues from rival groups, starting with scents, then calls, and finally live intruders. This experimental technique trades off complete natural ecological validity in exchange for greater control and direct testing of the hypotheses. We measured short‐term behavioral responses over a period of 1 h following presentation of each type of stimuli (totally 2 h on this day). On Days 4 and 5, we returned to the group for 2 h each day (one in the morning and one in the afternoon) to collect behavioral data, to test whether there were longer‐term impacts of the simulated conflict.

**FIGURE 1:**
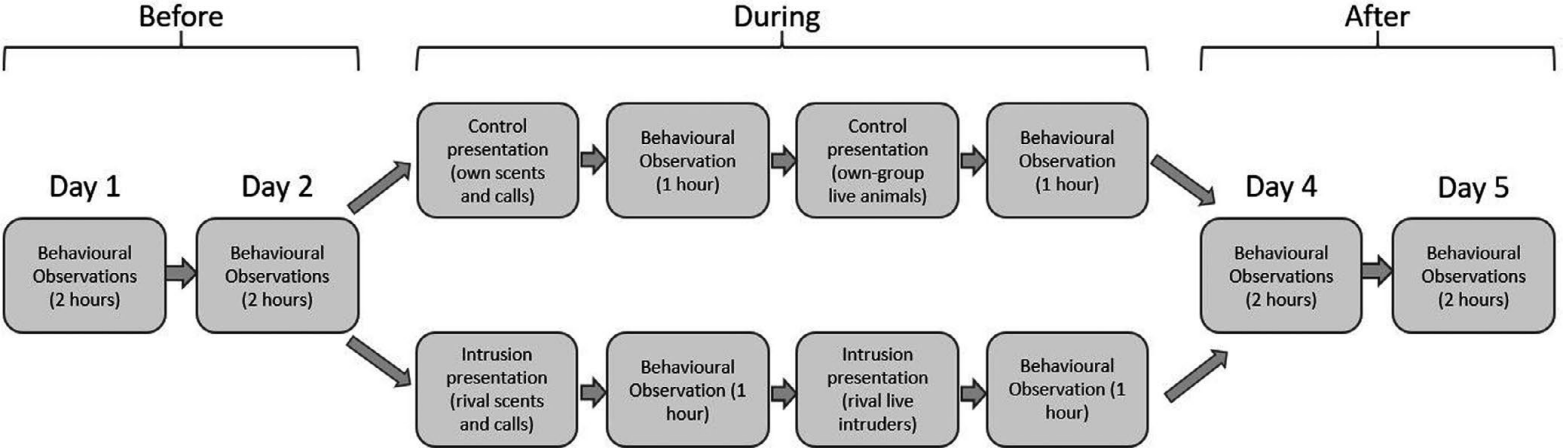
Summary of the experimental timeline – showing when behavioral observations and presentations of stimuli were performed

In addition, to confirm that the mongooses perceived outgroup stimuli as such, we carried out a set of “control” trials using equivalent “own‐group” stimuli, specifically scents, calls, and live individuals from their own group. Due to time and logistical constraints, we were forced to carry out control trials at a different time of year and using a different primary observer from intrusion trials (details in Table S1). This constraint meant that while “control” trials were useful to confirm that the mongooses reacted very differently to own versus other group stimuli, they were of limited use when comparing absolute levels of behavior before, during, and after the presentations between the experiment and “control” treatments. The appropriate “control” comparison for each type of trial was therefore the reference period before exposure to the stimuli, and so we analyzed behavior in the before versus after periods separately for intrusion and control trials. The timeline of the experiment is summarized in Figure 1.

### 2.3 Intrusion trials

We carried out repeated trials on each of five focal groups. Presentations to each focal group were separated by an average of 38 days (minimum 2 weeks) to reduce habituation of the mongooses to the stimuli. In total, we performed 22 intrusion trials and 22 own‐group control trials (see Table S1 for more information). The trials included 435.6 h of behavioral observations.

#### 2.3.1 Scents and war cries

Scents (which included feces, urine, and scent marks) from a neighboring group (usually the largest and/or closest neighboring group) were presented to the focal group on the morning of the presentation day (07:43–10:27 h). Neighboring groups were chosen because they pose the greatest risk to banded mongoose groups (Müller & Manser, 2007). These neighboring groups were chosen in advance of the study and remained the same throughout, except when two social groups merged, and new neighbors had to be chosen for the remaining trials. Feces, urine, and scent marks were collected from multiple individuals in the rival group as the group emerged from the den or at the first group latrine or marking site. Plastic sheets were laid out on the ground to encourage defecation, urination, and scent marking (as banded mongooses prefer marking and defecating on novel objects, particularly smooth materials like plastic), and to aid collection (these were easily washed thoroughly with soap and water between presentations). A standardized volume of feces and urine was used (100 × 137 mm zip lock bag). Samples were transferred as quickly as possible to the presentation site, and presented within 2 h of collection. To ensure that mongooses encountered these samples, they were arranged just ahead of the focal group on their travel path. The samples were arranged in a semicircle in an open area, with feces and urine placed around the sheets of plastic (spaced over 70–100 cm) as mongooses often use open patches for territorial marking (Müller & Manser, 2007).

We allowed the mongooses to explore the scents for 3 min, and then played a playback of “war cries” from the same rival group. Playbacks were started sooner if the mongooses began to move away from the area (mean time between scents and playback = 02:39 min, range = 01:38–04:03). We used a portable USB speaker (iHome IHM60) hidden in vegetation to play recordings of “war cries.” We used neighboring competitor groups, as these are known to be the greatest threat (Müller & Manser, 2007). War cries were recorded from the rival group during presentations of individuals (in traps) from the focal group. These presentations for recording purposes were conducted separately from intrusion trials and >1 week before playbacks to the focal group. War cries were recorded using an H1 Zoom recorder attached to a Sennheiser directional microphone (ME66/K6 and a wind buffer). We recorded calls from 2–3 m away, and cut them into 30‐s clips of vocalization, the amplitude of each clip was standardized using the normalize function in Audacity 2.1.2 to −1 dB (http://audacityteam.org). We only used each 30‐s playback clip once to prevent habituation of the mongooses to any of the recordings.

#### 2.3.2 Live intruders

On the afternoon of the same day (16:35–18:18 h), four adult male individuals from the rival group were trapped and presented to the focal group, following methods established in previous studies (Cant et al., 2002). The traps were washed with soap and water to reduce scents from any previous trapping events before the males were captured. No particular mongooses were selected based on social or dominance status, as all adult males could be considered a threat to rival groups (Cant et al., 2002). Animals in traps were covered with a black cloth to minimize stress at all times except during the 5‐min presentation. After 5 min the males were covered and returned to their own group, usually within 10–20 min. We used live intruders because we do not know what the pertinent signals to mongooses are in intergroup encounters, and live intruders provide multiple signals of immediate threat including visual, scent, and auditory signals. However, we designed the experiment to minimize any stress to the animals and paid close attention to the well‐being of the animal subjects throughout (see Section 2.6 below).

### 2.4 Control trials

Control presentations used scents collected from the focal group (the group being observed and presented to) early in the morning from multiple individuals, mirroring the intrusion trials. We then presented these focal group samples to the group once they moved to a new area (using a similar gap in time between collection and presentation as during intrusion trials). As in intrusion trials, a standardized volume of feces and urine was used (100 × 137 mm zip lock bag). “War cries” from any group including own group calls would be considered to indicate an outgroup threat as they would suggest a member of the group had detected an outgroup, so a non‐threatening call type was used as a control. We used “close calls” (a non‐threatening communication call between group members (Müller & Manser, 2008)) of the focal group to control for the presence of a speaker and a recording being played. These close calls were recorded as the focal group engaged in normal foraging, and when there was no other internal or external threat. Recordings were cut and standardized in the same way as in the intrusion trials. We presented four adult males from the focal group in traps, after they had been removed from the group for half an hour (and kept in a safe, shaded location). Removing the males for 30 min simultaneously allowed the group to return to normal behavior, in case they had been affected by trapping, and also simulated the entry of an animal not present in the pack immediately before the presentation, as in intruder trials.

### 2.5 Behavioral observations

Behavioral observations were carried out for 2 days preceding either a control or a simulated intrusion presentation (Before); on the day of the presentation (During); and for 2 days after the presentation (After). On each of the 5 days, we observed the focal group for 1 h in the morning (starting the search for mongooses at the same time, and beginning observations when the focal group was located, which ranged from 06:56 to 11:32 h, most started between 07:00 and 09:00 h and did not systematically vary among groups, days, or treatment types) and 1 h in the afternoon (starting the search at the same time, and beginning observations when the focal group were located, which ranged from 15:25 to 18:38 h, most started between 16:00 and 18:00 h and did not systematically vary among groups, days, or treatment types). We recorded grooming, aggression, collective scent marking, and collective alarm calling, as defined in Table 2, in an ad lib manner, recording all instances of these behaviors that involved adult and sub‐adult mongooses (>3 months old). On the day of the presentations, we carried out observations as soon as possible after the presentation ended for 1 h after each presentation. On the day of the presentation, we recorded the behavior in the first 5 min of the observation and then the next 55 min (5–60 min) to explore differences between immediate short‐term responses, and mid‐term responses to a simulated intrusion or “control” presentation. We chose a 5‐min window to measure this short‐term response as this mirrored the period for which the stimuli were present (~4 min for scents and calls, 5 min for live intruders), and mongooses typically left the presentation site a few minutes after stimuli were removed. During behavioral observations, no natural intergroup encounters were seen, but it is possible that there were unobserved encounters. In addition, we recorded immediate behavioral reactions to the stimuli as they were presented using a video camera. These videos were then analyzed by one observer to score the behavioral response of the group on a 6‐point ordinal scale (Table 3).

**TABLE 2:** Description of the behaviors of interest, recorded during behavioral observations

Behavior	Description
Grooming (or other affiliative) interaction	Grooming – one mongoose grooms another mongoose using their mouth, manipulating the fur with the teeth, the head moves in a distinctive backwards and forwards motion. One bout of grooming was defined as active grooming between the same pair of individuals with short breaks of no longer than 30 s of rest. If 30 s elapsed and the same pair began grooming again this was considered to be a second interaction. Grooming between multiple individuals switching from one partner to the other was recorded as one interaction per actor–recipient pair. Returning to a previous partner was not recorded as a separate interaction, unless 30 s of rest (no grooming of any partner) occurred. Nubbing – two mongooses perform “nubbing” behavior – a mutual genital sniff with raised tails which may also include marking each other and vocalizing (Preston et al., 2020)
Aggressive interaction	One mongoose is aggressive to another mongoose. This can include lunging, biting, growling, or snarling vocalizations, or physical displacement of another individual. Aggressive interactions happen over food resources, during mate guarding and as part of dominance interactions. One aggressive interaction was defined as aggression between the same pair of individuals with short breaks of no longer than 30 s between aggressive behaviors (e.g., lunging, vocalizing). (Preston et al., 2020)
Collective marking event	Three or more individuals mark the ground (or each other) with urine, feces, or scent marks (rubbing the anal or cheek glands along the surface). One individual marking or two individuals marking each other were not included as these behaviors are not considered collective
Collective alarm calling event	Two or more individuals simultaneously “alarm call” by standing in a bipedal stance observing the area with an alert and raised head, this may also be accompanied by alarm vocalizations – shrill, high‐pitched cries. This often recruits others to join the alarm calling event

**TABLE 3:** Description of the scoring of immediate behavioral reactions. Scores were recorded from video footage taken during the presentation of intrusion (rival scents, rival war cries, and rival intruders) and stimulus control (own scents, own close calls, and own individuals) stimuli

Score	Description
0	No reaction and no approach toward the stimulus by any individual
1	At least one individual approaches the stimulus with curiosity, but no alarm
2	At least one individual approaches the stimulus with curiosity, and a low level of alarm (less than 10 s of alarm calling or vigilance behavior)
3	Some (<50%) individuals mark, alarm call, and/or attack
4	Most (>50%) individuals mark, alarm call, and/or attack
5	All individuals mark, alarm call, and/or attack

### 2.6 Ethical note

All field research was carried out under permit from Uganda Wildlife Authority (Ref. COD/96/02) and Uganda National Council for Science and Technology (NS 591). Ethical Approval was received from the Ethical Committee of the University of Exeter and is in line with ASAB's Guidelines for the Treatment of Animals. Animals are trapped regularly as part of the long‐term data collection of the project. In this study, animals in traps were covered with a black cloth to minimize stress at all times except during the 5‐min presentation. Subjects were checked the next day in their own group (whether trapped or exposed to simulations) and there were no detectable effects on the behavior or health of any individuals used in the study.

### 2.7 Statistical analysis

#### 2.7.1 Behavior during exposure to stimuli

All statistical analysis was performed in R 3.2.2 (R Development Core Team, 2019). We analyzed immediate behavioral reaction scores (0–5, Table 3) measured from the videos using a cumulative link mixed model for ordinal regression using the *ordinal* package (Christensen, 2019). This analysis was to show statistically that mongooses reacted differently to “control” and intrusion trials, which was clear to a trained observer, to place the other results in context as intrusions and “controls” are not otherwise directly compared. Treatment type (“control” or intrusion) was the explanatory variable of interest, and stimulus type (scents and calls, or live intruders) and an interaction between treatment and stimulus type were also included in the model as explanatory factors. We included trial identity (categorical unique identity) as a random factor because of the repeated measure of the score between scents and calls, and live intruder presentations. Maximal models were fitted including all explanatory variables. We assessed the significance of each variable by removing the variable from the model and comparing the likelihood ratio of this model to the maximal model (Bates et al., 2015). Reported estimates and standard errors are from maximal models. We did not use a stepwise model reduction procedure because of problems associated with this method (Forstmeier & Schielzeth, 2011; Mundry & Nunn, 2009; Whittingham et al., 2006). Non‐significant interactions were removed from maximal models before main effects were tested (Engqvist, 2005). Post hoc tests were performed using the *emmeans* package in R, which calculates estimated marginal means from a model and contrasts them (Lenth, 2019); the Tukey method for multiple testing adjustment was used. We made pairwise comparisons between levels of significant interactions or main effects.

#### 2.7.2 Behavior after exposure to stimuli

Behavioral data were analyzed using generalized linear mixed models (GLMMs) using the *lme4* package (Bates & Maechler, 2009). The significance of each variable was tested in the same way as the video score analysis, but separate models were created for data from intrusion and “control” trials to avoid direct comparison between these two trial types. We analyzed the rate of grooming events, aggressive events, collective alarm calls, and collective marking events per hour as response variables in eight models (one for intrusion, and one for “control” trials for each behavioral response variable); each a Poisson model, including an offset of log(observation time) to account for counts being recorded over different length observation periods, using the same set of fixed and random effects. Explicitly, these effects were time point, group size, breeding status, rainfall, and an offset of time (to account for differential observation time), and random effects of experiment identity (as a categorical unique identity to account for repeated effects), group identity (which was removed from most models, see later explanation), or an observation‐level random effect (see full explanations in the next paragraphs). Most trials had the full 10 h of observations (Mean ± SE = 594 ± 2 min), but occasionally observations were cut short by bad weather, external interference, or animals returning to the den. For statistical analysis, we split observations of behavioral responses to the stimuli into four categories: the first two “baseline” days (Days 1–2), the first 5 min after presentation of the stimulus (0–5 min), next 55 min (5–60 min), and the next 2 days after the presentations (Days 4–5). We chose a 5‐min window to measure short‐term responses because this mirrored the period for which stimuli were presented (~4 min for scents and calls, 5 min for live intruders), and mongooses typically left the presentation site a few minutes after stimuli were removed. To control for variation in observation time between time points (i.e., Days 1–2, 0–5 min, 5–60 min, and Days 4–5), we included an offset of log(observation time) in the model as an additional fixed effect. An offset essentially models the variable as a rate, using the count and the exposure time.

To analyse changes in behavior in the periods before, during, and after the presentations, we did not differentiate between data from the two presentation stimulus types (scents/calls and intruder), since responses to these stimuli were not significantly different across the whole dataset (Tables S15‐S18, Figure S1A). The models contained time (Days 1–2, 0–5, 5–60, Days 4–5), the number of adult individuals present in the group during the observation day (babysitting individuals at the den were not included in this number), the breeding status of the group (estrus, pregnant, babysitting, escorting, and non‐breeding; see also Thompson, Marshall, Vitikainen, Young, et al. (2017)), and the mean rainfall from the last 30 days as explanatory variables. Group size (standardized) was included as a continuous variable to control for differences in group size on the count of behaviors, and to account for changes in group composition affecting the response. Breeding status was included because it may affect internal social dynamics within a group, for example, increased aggression rates during estrus as males mate‐guard females, and is known to affect frequency of intergroup encounters (Johnstone et al., 2020; Thompson, Marshall, Vitikainen, & Cant, 2017). Rainfall (standardized) was included as a proxy for resource and food availability, which could change competition levels and therefore aggression between individuals. The identity of the trial (due to the repeated measures nature of the experiment) was included as a random effect (Crawley, 2013), and an observation‐level random effect was used to address overdispersion in all models except grooming models (Harrison, 2014). Group identity was also initially included as a random effect, but was removed from the models as it did not explain any variation, and caused issues with singularity and overfitting of the models, in all models except grooming models. Random slopes were not included because there was no a priori reason to, and models would be exposed to overfitting (Grueber et al., 2011; Harrison, 2018). All final models were checked for overdispersion using the DHARMa package, and none were found to be either over‐ or underdispersed (Hartig, 2021).

## 3 RESULTS

In total, we performed 22 intrusion trials and 22 own‐group control trials, of which 6 intrusion and 6 control trials were performed in Group 1B, 6 intrusion and 6 control trials in Group 1H, 4 intrusion trials and 3 control trials in Group 11, 4 intrusion and 0 control trials in Group 2, and 2 intrusion and 7 control trials in Group 26. Group 2 dissolved before any successful control trials could take place, the female group members merged with Group 11 males to create Group 26 – and all remaining trials for these groups took place with group 26.

### 3.1 Behavior during exposure to stimuli

**FIGURE 2:**
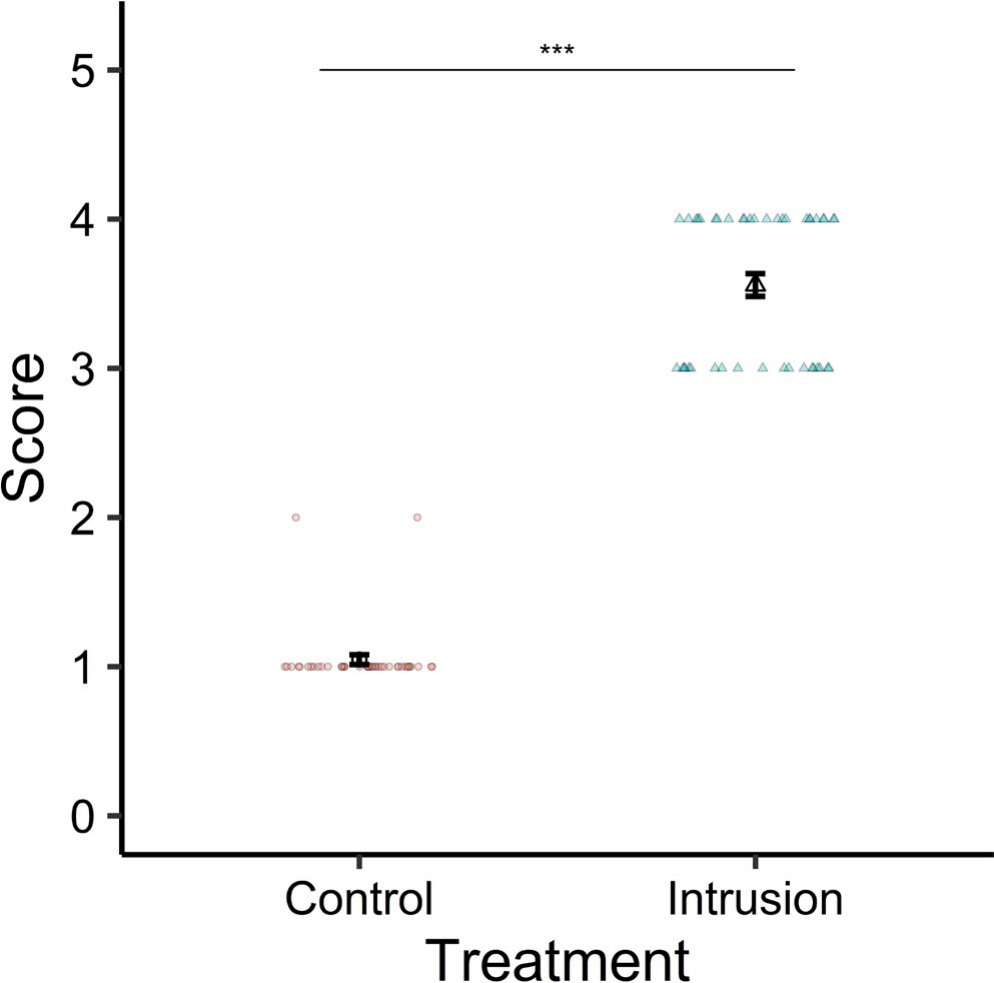
The immediate reaction score of banded mongoose groups to “control” versus intrusion stimuli. Scores ranged from 0 to 5, with 0 indicating no reaction or approach toward the stimuli, and 5 being the strongest reaction to the stimuli. Two types of stimuli were tested: scents combined with calls (“control” = own scents and close calls; intrusion = rival scents and war cries), or live intruders (“control” = 4 adult males from the focal group; intrusion = 4 adult males from the rival group). The immediate reaction to these types of stimuli was almost identical, so combined data are shown. Large black outline points show means from raw data, with standard error bars. Raw data are shown as small points. Intrusion trials are shown as triangles and “control” trials as circles. Note: due to logistical constraints, control and intrusion trials were run at different times of year (see Section 2)

Mongooses approached the stimuli in all intrusion and “control” trials. The animals responded in a very similar way to presentations of scents/calls and to live intruders; immediate reaction scores were not related to stimulus type (Estimate ± SE = 0.09 ± 0.59, $\chi_{1}^{2}$ = 0.03, *p* = .87). Reaction scores were much higher during intrusion trials than during “control” trials (Estimate ± SE = 47.55 ± 250.16, $\chi_{1}^{2}$ = 42.54, *p* < .001; Table S2, Figure 2), suggesting that mongooses clearly discriminate own from other group stimuli. However, these trials took place at different times of year, and under different observer conditions, so are not explicitly compared in any other analyses. They are only compared here to test whether mongooses are able to discriminate between the stimuli and react differently to them initially.

### 3.2 Behavior after exposure to stimuli

**FIGURE 3:**
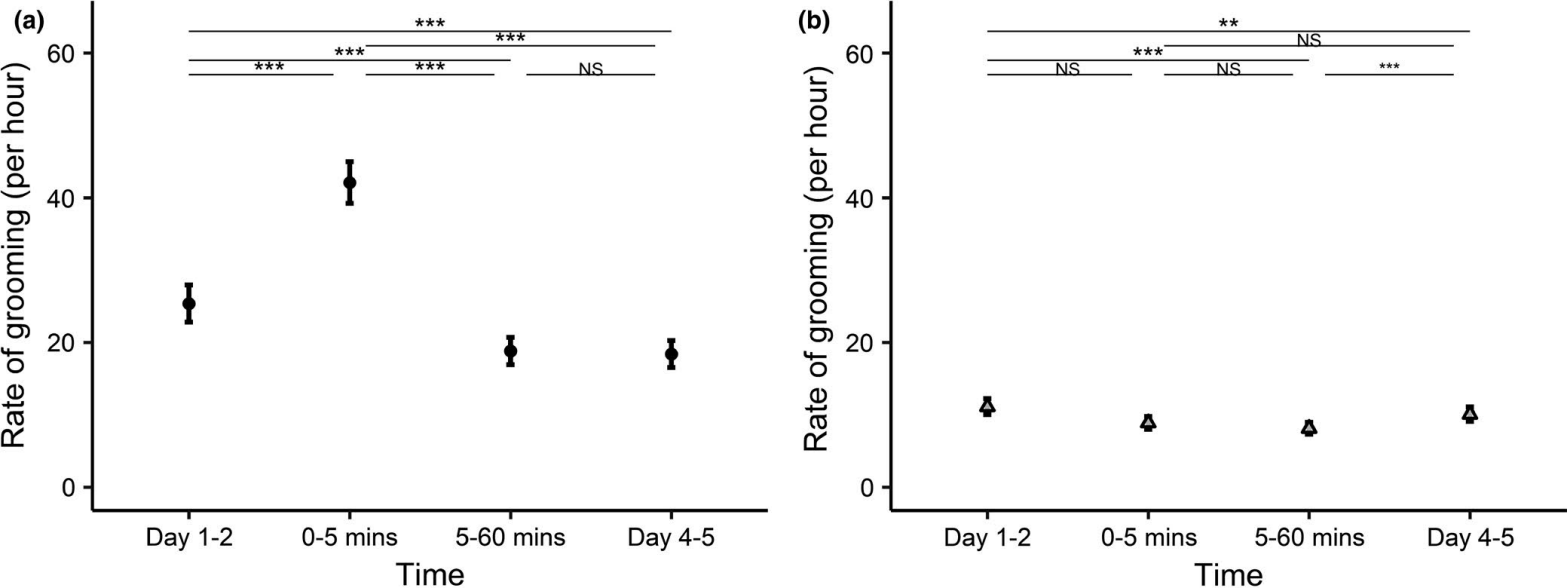
Dynamics of grooming. Rate of grooming (per hour) in (a) intrusion (filled circles) and (b) “control” (empty triangles) trials over the time course of the experiment, standard error bars are shown for each mean. Points show means from the GLMM ± SE. ***p < .001, **p < .01, *p < .05, NS *p* > .05; asterisks refer to post hoc pairwise comparison of means across all four time categories. Absolute values were analyzed separately, but are visualized to show patterns of the response (see Section 2)

Grooming behavior was affected by simulated territorial intrusions (Table S3: time point *χ* ^2^ = 302.36, *p* = <.001), increasing briefly from baseline grooming rates in the first 5 min after exposure (post hoc tests: Days 1–2 vs. 0–5 min: Estimate ± SE = −0.65 ± 0.07, *z* = −9.89, *p* < .001, Table S4), and then declining to below baseline level in the longer term between 5–60 min and in the 2 days afterwards (post hoc tests: Days 1–2 vs. 5–60 min: Estimate ± SE = 0.30 ± 0.04, *z* = 8.31, *p* < .001; Days 1–2 vs. Days 4–5: Estimate ± SE = 0.32 ± 0.03, *z* = 12.36, *p* < .001, Table S4, Figure 3). During intrusion trials, all pairwise post hoc comparisons between time points were significant (Table S4), except the comparison between 5–60 min and Days 4–5 in which grooming remained depressed compared to baseline rates (post hoc tests: 5–60 min vs. days 4–5: Estimate ± SE = 0.02 ± 0.04, *z* = 0.61, *p* = .93, Table S4, Figure 3). Absolute values between intrusion and control trials should not be compared directly here due to the trials being run at different times; patterns within trials should be compared.

In “control” trials grooming behavior was also affected by the presentations (Table S5: Time point *χ* ^2^ = 62.42, *p* < .001, Table S5), suggesting that some aspect of the experiment affected grooming behavior across time, or that grooming behavior is dynamic. In “control” trials, rates of grooming did not differ from baseline in the first 5 min after presentations (post hoc tests: Days 1–2 vs. 0–5 min: Estimate ± SE = 0.23 ± 0.11, *z* = 1.99, *p* = .19, Table S6), but was significantly lower than baseline levels in the longer term following presentations (post hoc tests: Days 1–2 vs. 5–60 min: Estimate ± SE = 0.31 ± 0.04, *z* = 7.64, *p* < .001; and Days 1–2 vs. Days 4–5: Estimate ± SE = 0.10 ± 0.03, *z* = 3.28, *p* = .01, Table S6). Grooming changed across time, but not in the same pattern as during intrusions, suggesting that intrusions had an impact above and beyond that of the experimental setup.

Additionally, larger groups groomed at a lower rate in intrusion trials (Table S3, group size estimate ± SE = −0.53 ± 0.06, $\chi_{1}^{2}$ = 82.18, *p* < .001), but we did not find this pattern in “control” trials (Table S5). Rainfall was negatively associated with rates of grooming in both “control” and intrusion trials (Table S3 intrusion trials, rainfall estimate ± SE = −0.23 ± 0.05, $\chi_{1}^{2}$ = 18.53, *p* < .001; Table S5 “control” trials, rainfall estimate ± SE = −0.39 ± 0.10, $\chi_{1}^{2}$ = 15.49, *p* < .001). Breeding status did not affect grooming behavior in either intrusion or “control” trials (Tables S3 and S5).

Rates of aggression in both intrusion and “control” trials varied in the same pattern across time (Table S7 intrusion trials, time point: *χ* ^2^ = 14.91, *p* < .001; Table S9 “control” trials, time point: *χ* ^2^ = 16.77, *p* < .001), and post hoc tests showed a decrease in rates of aggression during the first 5 min compared to baseline levels (Table S8 intrusion trials, post hoc tests, Days 1–2 vs. 0–5 min: Estimate ± SE = 0.54 ± 0.20, *z* = 2.72, *p* = .03, Table S10 “control” trials, post hoc tests, Days 1–2 vs. 0–5 min: Estimate ± SE = 0.89 ± 0.30, *z* = 2.99, *p* = .01), and higher aggression rates in Days 4 and 5 compared to 0–5 min as levels return to baseline (Table S8 intrusion trials, post hoc tests, 0–5 min vs. Days 4–5: Estimate ± SE = −0.53 ± 0.20, *z* = −2.70, *p* = .04; Table S10 “control” trials, post hoc tests, 0–5 min vs. Days 4–5: Estimate ± SE = −1.03 ± 0.30, *z* = −3.428, *p* = .003).

Rates of aggression were also affected by group size. Larger groups were more aggressive in both intrusion and “control” trials (Table S7 intrusion trials, group size estimate ± SE = 0.56 ± 0.12, $\chi_{1}^{2}$ = 14.91, *p* < .001; Table S9 “control” trials, group size estimate ± SE = 0.43 ± 0.14, $\chi_{1}^{2}$ = 7.92, *p* < .01). Breeding status and rainfall did not affect aggression rates (Tables S7 and S9).

There was no change in rates of collective scent marking or alarm calling over time points in either intrusion or “control” trials (Tables S11, S12, S14, and S15). Other predictors (breeding status, rainfall, and group size) also had no significant effect (Tables S11, S12, S14, and S15) except for rates of collective scent marking in “control” trials which increased with rainfall (Table S12, rainfall estimate ± SE = 0.26 ± 0.12, $\chi_{1}^{2}$ = 4.47, *p* = .03) and were affected by breeding status (see Table S13 for details).

## 4 DISCUSSION

Banded mongooses showed strong immediate behavioral reactions to simulated intergroup encounters during the presentation of the stimuli. Moreover, simulated encounters resulted in higher rates of grooming in the first 5 min and lower rates of grooming over the rest of the subsequent hour and 2 days compared to baseline rates. However, the longer‐term change in grooming must be interpreted with caution, as grooming was also lower in these time periods during “control” trials. Simulated intergroup encounters had no effect above and beyond “control” patterns on aggression rates or two other collective behaviors, scent marking and alarm calling, in either the short or longer term.

The initial increase in grooming we observed after a simulated intergroup encounter matches increases in similar experiments in other social vertebrates (see Table 1); however, the decrease in grooming we observed in the longer term between 5–60 min and the following 2 days contrasts with these previous studies. In other social animals, increases in post‐conflict within‐group affiliative behavior have been recorded in both experimental simulations and observations of natural encounters (Table 1). Some of these studies show increased grooming at similar short timescales of around 10 or 20 min, whereas others suggest increased grooming for 1 h or even 1 day (Table 1). In these studies, elevated rates of grooming (or other affiliative behaviors such as social contacts (Birch et al., 2019; Thompson et al., 2020) or soft “bumps” (Bruintjes et al., 2015)) are taken as measures of increased social cohesion, or strengthened social relationships. Other studies have found no change in rates of grooming (Nunn & Deaner, 2004). Our finding that grooming increased initially in the short term, then subsequently declined below baseline levels in the longer term, suggests that a simulated intrusion may impact grooming behavior long after the threat has passed, but further study to confirm whether this is the case in this and other species is needed. Although the dynamics of grooming response were very different in intrusion and “control” trials, even “control” trials showed changes in social behavior in the post‐period. This finding highlights that there may be subtle and unanticipated changes in behavior as a result of experimental presentations of stimuli or apparatus. For example, in this study, changes in long‐term behavior in “controls” could conceivably be linked to the temporary removal of four group members for the “control” presentations, and changes in grooming or other behaviors in the medium term on their return to the group when grooming was lower than before presentations.

Our study is a rare example of intergroup conflict leading to a potential decrease in grooming (see Yi et al., 2020, for a similar result in gibbons), which highlights questions about the function and context of grooming behavior in different species and how we might expect social animals to respond to intergroup threats. Grooming is a key measure of primate relationships, and has been proposed to serve as a commodity in biological markets, exchanged, for example, for reciprocal grooming, resources, or rank‐related benefits (Balasubramaniam & Berman, 2017; Barrett et al., 1999, but see Sánchez‐Amaro & Amici, 2015). In vervet monkeys, grooming by females between "bouts” during intergroup contests may encourage males to increase their investment in group defense (Arseneau‐Robar et al., 2016). By contrast, in banded mongooses grooming is usually expressed during periods of rest and play, not in the face of a threat. Reduced grooming after exposure to intergroup threats may therefore reflect increased stress, vigilance, or other defensive activities, after an initial short‐lived increase for social bonding or stress relief. In gibbons, reduced grooming between pairs after intergroup encounters is suggested to be a byproduct of increased foraging, after a reduction in foraging time during encounters (Yi et al., 2020). In general, behaviors such as grooming (and, potentially, aggression) that may appear similar in different species may function differently and be expressed in different contexts, depending on ecology, social structure, and sensory abilities.

We found a brief reduction in within‐group aggression after an intergroup threat, but this was not limited to intrusion trials. As with grooming behavior, this effect might reflect other changes in behavior resulting from the experiment. Aggression in banded mongooses often occurs in the context of foraging, but mongooses stopped foraging during exposure to stimuli, and left the site of the presentations shortly afterwards. Importantly, we observed the same pattern across time periods, and a similar decline in aggression in both intrusion and “control” trials, suggesting that the effect on aggression is likely to reflect a behavioral response to the experimental apparatus, not to a simulated intergroup encounter per se, or to another unmeasured variable. In other systems, the effect of intergroup conflict on post‐conflict aggression is mixed. In some species, within‐group aggression is elevated in the post‐conflict period, for example, in capuchin monkeys (Polizzi di Sorrentino et al., 2012) and bonnet macaques (Cooper et al., 2004), and in others, it is lower, for example, chimpanzees (Brooks et al., 2021). By contrast, intergroup encounters resulted in no change in within‐group aggression in the cichlid (Bruintjes et al., 2015), dwarf mongooses (Morris‐Drake et al., 2019), or ring‐tailed lemurs (Nunn & Deaner, 2004). To interpret this variation in observed patterns requires information about the potential function of aggressive behavior. Is aggression related to resource competition, or to social status or reproductive shares? Which individuals are the actors and which are the recipients, and how does the behavior of each change after aggressive acts? Studies investigating the function of aggression and its effects on participation in intergroup conflict (e.g., Arseneau‐Robar et al., 2016) may help to explain some of the diversity in average levels of post‐conflict behavior observed in different systems.

Most previous studies focus on the same day responses to intergroup conflict, and with the exception of some studies of ranging behavior, territory use, and group modularity (Crofoot, 2013; Markham et al., 2012; Radford & Fawcett, 2014, Samuni et al., 2020), few studies have attempted to measure longer‐term impacts (see Table 1). Radford and Fawcett (2014) found that intergroup conflicts in green wood hoopoes resulted in increased preening behavior at the roost, several hours later, and Samuni et al. (2020) found changes in intragroup behavior and cohesion on days when intergroup intrusions occurred, versus those when they did not, suggesting that there may be durable behavioral impacts of intergroup conflicts. But how long should we expect responses to natural or simulated intergroup encounters to last? Time invested in vigilance or defensive behaviors usually comes at a cost to foraging or other fitness‐related activities such as territory defense, so we might expect the durability of behavioral responses to a single outgroup threat stimulus to be related to the frequency of intergroup encounters. Moreover, in systems in which intergroup encounters are already common, it may be difficult to isolate the impacts of a single intergroup threat stimulus. Intergroup encounters in banded mongooses occur frequently (mean encounter rate per group = 0.8 per week (non‐estrus periods) to 2.9 per week (group estrus); data from 12 groups (Cant et al., 2002). By comparison, the average rate of natural intergroup encounters in a recent study of African wild dogs was once every 7 weeks (Jordan et al., 2017). It is possible that natural intergroup encounters occurred during the study and affected longer term behavior, which may have impacted these results. If grooming and aggression rates before a presentation are already high in banded mongooses as a result of recent natural encounters, a single simulated intergroup encounter may cause little change in the level of grooming or aggression seen within the group, despite affecting other behaviors. This might explain why the expected increase in grooming interactions is only seen for 5 min after a simulated intrusion, and longer‐term changes are less clear. Heightened baseline behavior may also help to explain why there is no detected change in scent marking or alarm calling behavior during or after the simulated intrusion. Studying populations of the same species that experience different overall levels of intergroup conflict could help to assess how responses to the same manipulation vary with background levels of conflict.

Importantly, although we detect short‐ and potential longer‐term changes in the average levels of grooming within the group, this result may mask more subtle changes in intragroup interactions that arise from within‐group heterogeneity. It is well documented that different types of individuals contribute to intergroup conflicts to different degrees (Arseneau et al., 2015; Boydston et al., 2001; Koch et al., 2016; Mares et al., 2012; Muller & Mitani, 2002; Radford, 2008a; Van Belle et al., 2014; Wilson et al., 2014). Males and females often have different costs and benefits associated with participation in intergroup encounters, and therefore behave differently (Boydston et al., 2001; Koch et al., 2016; Mares et al., 2012; Muller & Mitani, 2002; Wilson et al., 2014). Dominant and subordinate individuals also experience different costs, which can influence their involvement (Arseneau et al., 2015; Koch et al., 2016; Radford, 2008a; Van Belle et al., 2014). In green wood hoopoes, for example, allo‐preening by dominant individuals was directed toward subordinates after conflicts (Radford, 2008b). In a recent paper based on the same experiment reported here, we used social network analysis to investigate whether there were more subtle impacts of outgroup threats on patterns of social interaction among males and females (Preston et al., 2020). There were no durable impacts of simulated intergroup conflict on the level of social cohesion as measured by eigenvector centrality in the grooming network (Preston et al., 2020). However, we found that male‐to‐male, male‐to‐female, and female‐to‐male grooming strength decreased in the 2 days after simulated intrusions. This supports our interpretation of grooming rates being reduced, which is difficult to conclude from this study alone, and shows which relationships are affected.

Despite the collective, and potentially cooperative, nature of scent marking and alarm calling, neither of these behaviors were affected by simulated intergroup intrusions. Alarm calling and vigilance could be beneficial in avoiding future contests, and mongooses clearly respond to the scent marks, war cries, and presence of neighbors, as shown by their strong initial reaction to these stimuli in this experiment. However, this does not seem to have a lasting effect on their behavior, even during the first 5 min after stimuli are removed. Neither marking nor vigilance has been studied much in the context of intergroup encounters, but one recent study found increasing levels of sentinel behavior in dwarf mongooses in response to simulated intergroup encounters (Morris‐Drake et al., 2019), and female marmosets increase scent marking when exposed to outgroup females (Schaffner & French, 1997).

One additional conclusion from this experiment is that the use of live intruders is not necessary in future experiments which investigate intergroup conflict in banded mongooses. Analysis revealed that there was no difference in the behavioral responses to scents/marks and playbacks in combination, or to live intruders (Tables S16‐S19, Figure S1). Live intruders were used in order to ensure that mongooses reacted to these simulated intruders and cover many sensory modes by which mongooses might detect mongooses from other groups (visual, scent, sound, etc.) but scent and sound appear to be enough to adequately simulate intergroup encounters in this species.

In conclusion, banded mongooses showed short‐lived increases in grooming in response to simulated intergroup encounters. Our findings raise questions about the extent to which behaviors used to measure social cohesion (e.g., grooming) are comparable across species. These behaviors are species and context dependent, and using them to define social cohesion a priori can risk misinterpretation of these behaviors. The potentially fleeting nature of behavioral impacts highlights the disparity between observed individual behavioral responses, which are inherently ephemeral and dynamic, and the static assumptions of population genetic and game theoretic models of intergroup conflict and cooperation. This is an area of research where empirical studies have started to reveal fascinating variation in behavior which current theory is not well suited to explain. Here, we start to bridge this gap, showing possible changes to affiliative behavior in the longer term, not in the direction predicted, but these should be confirmed or refuted by further work due to similar patterns in “control” trials which took place at a different time, to confirm these results hold in a fully counter‐balanced design. Further research is needed to truly bridge this gap between empirical and theoretical studies, and to evaluate longer‐term consequences of intergroup conflict for social relationships, survival, and fitness.

## CONFLICT OF INTEREST

None of the authors have any conflict of interest.

## AUTHOR CONTRIBUTIONS

**Elizabeth F. R. Preston:** Conceptualization (equal); data curation (equal); formal analysis (equal); investigation (equal); methodology (equal); visualization (equal); writing – original draft (lead); writing – review and editing (lead). **Faye J. Thompson:** Conceptualization (equal); formal analysis (supporting); funding acquisition (equal); project administration (equal); writing – review and editing (equal). **Solomon Kyabulima:** Data curation (supporting); methodology (supporting); resources (supporting); writing – review and editing (supporting). **Darren P. Croft:** Funding acquisition (equal); supervision (equal). **Michael A. Cant:** Conceptualization (equal); funding acquisition (equal); project administration (equal); supervision (equal); writing – original draft (supporting); writing – review and editing (equal).

## Supporting information

Appendix S1

Data S1

Data S2

## Data Availability

Data are available on Dryad at https://doi.org/10.5061/dryad.c2fqz619g.
