# Supplementary material for: The dynamics of social cohesion in response to simulated intergroup conflict in banded mongooses
Source: Ecol Evol. 2021 Dec 20;11(24):18662–75. doi: 10.1002/ece3.8475 (PMC8717285; doi:10.1002/ece3.8475)
Supplement: Supplementary file 1 — Appendix S1 [file ECE3-11-18662-s002.docx]

Supplementary Information

Table S1 – Distribution of experimental trials across focal mongoose groups.

| **Group** | **Intrusion Trials** | **Dates (of presentation – Day 3 of trial)** | **Control Trials** | **Dates (of presentation – Day 3 of trial)** |
| --- | --- | --- | --- | --- |
| 1B | 6 | \| 20/04/2016 \| \| --- \| \| 18/05/2016 \| \| 05/10/2016 \| \| 26/10/2016 \| \| 29/03/2017 \| \| 03/05/2017 \| | 6 | \| 29/06/2016 \| \| --- \| \| 28/07/2016 \| \| 17/08/2016 \| \| 15/03/2017 \| \| 21/06/2017 \| \| 19/07/2017 \| |
| 1H | 6 | \| 09/05/2016 \| \| --- \| \| 28/09/2016 \| \| 09/11/2016 \| \| 23/11/2016 \| \| 19/04/2017 \| \| 10/05/2017 \| | 6 | \| 06/07/2016 \| \| --- \| \| 03/08/2016 \| \| 24/08/2016 \| \| 22/02/2017 \| \| 28/06/2017 \| \| 26/07/2017 \| |
| 11 | 4 | \| 25/04/2016 \| \| --- \| \| 11/05/2016 \| \| 12/10/2016 \| \| 02/11/2016 \| | 3* | \| 22/06/2016 \| \| --- \| \| 13/07/2016 \| \| 10/08/2016 \| |
| 2 | 4 | \| 17/05/2016 \| \| --- \| \| 19/10/2016 \| \| 16/11/2016 \| \| 30/11/2016 \| | 0* |  |
| 26 | 2 | \| 22/03/2017 \| \| --- \| \| 26/04/2017 \| | 7* | \| 25/01/2017 \| \| --- \| \| 16/02/2017 \| \| 08/03/2017 \| \| 24/05/2017 \| \| 14/06/2017 \| \| 12/07/2017 \| \| 02/08/2017 \| |

* Group 2 dissolved before any successful control trials could take place. The female group members merged with group 11 males to create group 26 – all remaining trials for these groups took place with group 26.

Table S2 – Model predicting the immediate behavioural reaction score of mongooses following a presentation. Model was fitted using a cumulative link mixed model for ordinal regression, (CLMM, N = 86 videos from 44 trials). Significant terms are given in bold.

| Parameter | | Estimate | Standard Error | Χ^2^ | P |
| --- | --- | --- | --- | --- | --- |
| Treatment type | Intrusion | 0.00 | 0.00 | **83.01** | **<0.001** |
|  | Control | 47.55 | 250.16 |  |  |
| Stimulus type | Live intruders | 0.00 | 0.00 | 0.03 | 0.87 |
|  | Scents and calls | 0.09 | 0.59 |  |  |

Table S3 – Model predicting the frequency of grooming interactions during intrusion trials. Model was fitted using a Poisson error structure and a log link function, with observation times as an offset term, group ID and trial ID as random intercepts, and an observation-level random intercept (GLMM, N = 22 trials in 5 groups). Significant terms are given in bold.

| Parameter | | Estimate | Standard Error | Χ^2^ | P |
| --- | --- | --- | --- | --- | --- |
| Intercept | | -0.54 | 0.42 |  |  |
| Time point | Day 1-2 | 0.00 | 0.00 | **302.36** | **<0.001** |
|  | 0-5 minutes | 0.65 | 0.07 |  |  |
|  | 5-60 minutes | -0.30 | 0.04 |  |  |
|  | Day 4-5 | -0.32 | 0.03 |  |  |
| Rainfall | | -0.23 | 0.05 | **18.53** | **<0.001** |
| Group Size | | -0.53 | 0.06 | **82.18** | **<0.001** |
| Breeding status | Babysitting | 0.00 | 0.00 | 5.16 | 0.27 |
|  | Escorting | -0.14 | 0.32 |  |  |
|  | Non-breeding | -0.07 | 0.33 |  |  |
|  | Oestrus | -0.52 | 0.45 |  |  |
|  | Pregnant | 0.41 | 0.29 |  |  |

Table S4 – Post-hoc tests (contrasts between estimated marginal means) to determine differences in the frequency of grooming in intrusion trials between time points.

|  | Estimate | Standard Error | z | P |
| --- | --- | --- | --- | --- |
| Day 1-2 vs 0-5 minutes | **-0.65** | **0.07** | **-9.89** | **<0.001** |
| Day 1-2 vs 5-60 minutes | **0.30** | **0.04** | **8.31** | **<0.001** |
| Day 1-2 vs Day 4-5 | **0.32** | **0.03** | **12.36** | **<0.001** |
| 0-5 minutes vs 5 to 60 minutes | **0.95** | **0.07** | **13.44** | **<0.001** |
| 0-5 minutes vs Day 4-5 | **0.97** | **0.07** | **14.61** | **<0.001** |
| 5-60 minutes vs Day 4-5 | 0.02 | 0.04 | 0.61 | 0.93 |

Table S5 – Model predicting the frequency of grooming interactions during ‘control’ trials. Model was fitted using a Poisson error structure and a log link function, with observation times as an offset term, group ID and trial ID as random intercepts, and an observation-level random intercept (GLMM, N = 22 trials in 4 groups). Significant terms are given in bold.

| Parameter | | Estimate | Standard Error | Χ^2^ | P |
| --- | --- | --- | --- | --- | --- |
| Intercept | | -1.27 | 0.25 |  |  |
| Time point | Day1-2 | 0.00 | 0.00 | **62.42** | **<0.001** |
|  | 0-5 minutes | -0.23 | 0.11 |  |  |
|  | 5-60 minutes | -0.31 | 0.04 |  |  |
|  | Day4-5 | -0.10 | 0.03 |  |  |
| Rainfall | | -0.39 | 0.10 | **15.49** | **<0.001** |
| Group Size | | 0.09 | 0.19 | 0.20 | 0.65 |
| Breeding status | Babysitting | 0.00 | 0.00 | 4.88 | 0.30 |
|  | Escorting | 0.19 | 0.28 |  |  |
|  | Non-breeding | -0.14 | 0.24 |  |  |
|  | Oestrus | -0.90 | 0.54 |  |  |
|  | Pregnant | -0.02 | 0.30 |  |  |

Table S6 – Post-hoc tests (contrasts between estimated marginal means) to determine differences in the frequency of grooming in ‘control’ trials between time points.

|  | Estimate | Standard Error | z | P |
| --- | --- | --- | --- | --- |
| Day 1-2 vs 0-5 minutes | 0.23 | 0.11 | 1.99 | 0.19 |
| Day 1-2 vs 5-60 minutes | **0.31** | **0.04** | **7.64** | **<0.001** |
| Day 1-2 vs Day 4-5 | **0.10** | **0.03** | **3.28** | **0.01** |
| 0-5 minutes vs 5 to 60 minutes | 0.09 | 0.11 | 0.75 | 0.88 |
| 0-5 minutes vs Day 4-5 | -0.12 | 0.11 | -1.12 | 0.68 |
| 5-60 minutes vs Day 4-5 | **-0.21** | **0.04** | **-5.16** | **<0.001** |

Table S7 – Model predicting the frequency of aggressive interactions during intrusion trials. Model was fitted using a Poisson error structure and a log link function, with observation times as an offset term, and trial ID as a random intercept, and an observation-level random intercept (GLMM, N = 22 trials in 5 groups). Significant terms are given in bold.

| Parameter | | Estimate | Standard Error | Χ^2^ | P |
| --- | --- | --- | --- | --- | --- |
| Intercept | | -0.86 | 0.24 |  |  |
| Time point | Day 1-2 | 0.00 | 0.00 | **8.91** | **0.03** |
|  | 0-5 minutes | -0.54 | 0.20 |  |  |
|  | 5-60 minutes | -0.04 | 0.14 |  |  |
|  | Day 4-5 | 0.00 | 0.13 |  |  |
| Rainfall | | -0.06 | 0.14 | 0.20 | 0.65 |
| Group Size | | 0.56 | 0.12 | **14.91** | **<0.001** |
| Breeding status | Babysitting | 0.00 | 0.00 | 2.45 | 0.65 |
|  | Escorting | -0.21 | 0.29 |  |  |
|  | Non-breeding | -0.34 | 0.32 |  |  |
|  | Oestrus | -0.62 | 0.44 |  |  |
|  | Pregnant | -0.30 | 0.31 |  |  |

Table S8 – Post-hoc tests (contrasts between estimated marginal means) to determine differences in the frequency of aggression in intrusion trials between time points.

|  | Estimate | Standard Error | z | P |
| --- | --- | --- | --- | --- |
| Day 1-2 vs 0-5 minutes | **0.54** | **0.20** | **2.72** | **0.03** |
| Day 1-2 vs 5-60 minutes | 0.04 | 0.14 | 0.32 | 0.99 |
| Day 1-2 vs Day 4-5 | 0.00 | 0.13 | 0.04 | 1.00 |
| 0-5 minutes vs 5 to 60 minutes | -0.50 | 0.20 | -2.45 | 0.07 |
| 0-5 minutes vs Day 4-5 | **-0.53** | **0.20** | **-2.70** | **0.04** |
| 5-60 minutes vs Day 4-5 | -0.04 | 0.14 | -0.28 | 0.99 |

Table S9 – Model predicting the frequency of aggressive interactions during ‘control’ trials. Model was fitted using a Poisson error structure and a log link function, with observation times as an offset term, and trial ID as a random intercept, and an observation-level random intercept (GLMM, N = 22 trials in 4 groups). Significant terms are given in bold.

| Parameter | | Estimate | Standard Error | Χ^2^ | P |
| --- | --- | --- | --- | --- | --- |
| Intercept | | -2.08 | 0.28 |  |  |
| Time point | Day 1-2 | 0.00 | 0.00 | **16.77** | **<0.001** |
|  | 0-5 minutes | -0.90 | 0.30 |  |  |
|  | 5-60 minutes | -0.34 | 0.20 |  |  |
|  | Day 4-5 | 0.14 | 0.19 |  |  |
| Rainfall | | -0.09 | 0.19 | 0.23 | 0.63 |
| Group Size | | 0.43 | 0.14 | **7.92** | **0.005** |
| Breeding status | Babysitting | 0.00 | 0.00 | 8.54 | 0.07 |
|  | Escorting | 0.78 | 0.31 |  |  |
|  | Non-breeding | 0.23 | 0.30 |  |  |
|  | Oestrus | -0.85 | 0.64 |  |  |
|  | Pregnant | 0.15 | 0.38 |  |  |

Table S10 – Post-hoc tests (contrasts between estimated marginal means) to determine differences in the frequency of aggression in ‘control’ trials between time points.

|  | Estimate | Standard Error | z | P |
| --- | --- | --- | --- | --- |
| Day 1-2 vs 0-5 minutes | **0.89** | **0.30** | **2.99** | **0.01** |
| Day 1-2 vs 5-60 minutes | 0.34 | 0.20 | 1.70 | 0.32 |
| Day 1-2 vs Day 4-5 | -0.14 | 0.19 | -0.72 | 0.89 |
| 0-5 minutes vs 5 to 60 minutes | -0.56 | 0.30 | -1.86 | 0.25 |
| 0-5 minutes vs Day 4-5 | **-1.03** | **0.30** | **-3.428** | **0.003** |
| 5-60 minutes vs Day 4-5 | -0.48 | 0.20 | -2.38 | 0.08 |

Table S11 – Model predicting the frequency of collective scent marking during intrusion trials. Model was fitted using a Poisson error structure and a log link function, with observation times as an offset term, and trial ID as a random intercept, and an observation-level random intercept (GLMM, N = 22 trials in 5 groups). Significant terms are given in bold.

| Parameter | | Estimate | Standard Error | Χ^2^ | P |
| --- | --- | --- | --- | --- | --- |
| Intercept | | -2.87 | 0.22 |  |  |
| Time point | Day 1-2 | 0.00 | 0.00 | 3.81 | 0.28 |
|  | 0-5 minutes | 0.52 | 0.26 |  |  |
|  | 5-60 minutes | 0.13 | 0.16 |  |  |
|  | Day 4-5 | 0.14 | 0.15 |  |  |
| Rainfall | | -0.06 | 0.13 | 0.21 | 0.65 |
| Group Size | | -0.08 | 0.10 | 0.72 | 0.40 |
| Breeding status | Babysitting | 0.00 | 0.00 | 2.06 | 0.73 |
|  | Escorting | -0.31 | 0.26 |  |  |
|  | Non-breeding | -0.03 | 0.28 |  |  |
|  | Oestrus | -0.01 | 0.37 |  |  |
|  | Pregnant | -0.27 | 0.27 |  |  |

Table S12 – Model predicting the frequency of collective scent marking during ‘control’ trials. Model was fitted using a Poisson error structure and a log link function, with observation times as an offset term, and trial ID as a random intercept, and an observation-level random intercept (GLMM, N = 22 trials in 4 groups). Significant terms are given in bold.

| Parameter | | Estimate | Standard Error | Χ^2^ | P |
| --- | --- | --- | --- | --- | --- |
| Intercept | | -2.95 | 0.17 |  |  |
| Time point | Day 1-2 | 0.00 | 0.00 | 2.21 | 0.53 |
|  | 0-5 minutes | -0.34 | 0.33 |  |  |
|  | 5-60 minutes | -0.14 | 0.13 |  |  |
|  | Day 4-5 | -0.04 | 0.11 |  |  |
| Rainfall | | 0.26 | 0.12 | **4.47** | **0.03** |
| Group Size | | 0.15 | 0.09 | 2.64 | 0.10 |
| Breeding status | Babysitting | 0.00 | 0.00 | **11.58** | **0.02** |
|  | Escorting | 0.57 | 0.20 |  |  |
|  | Non-breeding | 0.05 | 0.20 |  |  |
|  | Oestrus | 0.46 | 0.39 |  |  |
|  | Pregnant | 0.62 | 0.24 |  |  |

Table S13 – Post-hoc tests (contrasts between estimated marginal means) to determine differences in the frequency of collective scent marking in ‘control’ trials at different breeding statuses.

|  | Estimate | Standard Error | z | P |
| --- | --- | --- | --- | --- |
| Babysitting vs Escorting | **-0.57** | **0.20** | **-2.86** | **0.03** |
| Babysitting vs Non-breeding | -0.05 | 0.20 | -0.24 | 1.00 |
| Babysitting vs Oestrus | -0.46 | 0.39 | -1.17 | 0.77 |
| Babysitting vs Pregnant | -0.62 | 0.24 | -2.55 | 0.08 |
| Escorting vs Non-breeding | 0.52 | 0.21 | 2.54 | 0.08 |
| Escorting vs Oestrus | 0.11 | 0.38 | 0.29 | 1.00 |
| Escorting vs Pregnant | -0.05 | 0.24 | -0.22 | 1.00 |
| Non-breeding vs Oestrus | -0.41 | 0.38 | -1.08 | 0.81 |
| Non-breeding vs Pregnant | -0.57 | 0.21 | -2.69 | 0.06 |
| Oestrus vs Pregnant | -0.16 | 0.35 | -0.47 | 0.99 |

Table S14 – Model predicting the frequency of collective alarm calling during intrusion trials. Model was fitted using a Poisson error structure and a log link function, with observation times as an offset term, and trial ID as a random intercept, and an observation-level random intercept (GLMM, N = 22 trials in 5 groups). Significant terms are given in bold.

| Parameter | | Estimate | Standard Error | Χ^2^ | P |
| --- | --- | --- | --- | --- | --- |
| Intercept | | -2.90 | 0.26 |  |  |
| Time point | Day 1-2 | 0.00 | 0.00 | 3.14 | 0.37 |
|  | 0-5 minutes | 0.39 | 0.26 |  |  |
|  | 5-60 minutes | 0.18 | 0.13 |  |  |
|  | Day 4-5 | 0.06 | 0.12 |  |  |
| Rainfall | | -0.05 | 0.15 | 0.12 | 0.72 |
| Group Size | | -0.17 | 0.12 | 1.71 | 0.19 |
| Breeding status | Babysitting | 0.00 | 0.00 | 0.82 | 0.94 |
|  | Escorting | -0.23 | 0.33 |  |  |
|  | Non-breeding | -0.25 | 0.35 |  |  |
|  | Oestrus | 0.03 | 0.47 |  |  |
|  | Pregnant | -0.17 | 0.34 |  |  |

Table S15 – Model predicting the frequency of collective alarm calling during ‘control’ trials. Model was fitted using a Poisson error structure and a log link function, with observation times as an offset term, and trial ID as a random intercept, and an observation-level random intercept (GLMM, N = 22 trials in 4 groups). Significant terms are given in bold.

| Parameter | | Estimate | Standard Error | Χ^2^ | P |
| --- | --- | --- | --- | --- | --- |
| Intercept | | -3.06 | 0.23 |  |  |
| Time point | Day 1-2 | 0.00 | 0.00 | 0.92 | 0.82 |
|  | 0-5 minutes | 0.03 | 0.34 |  |  |
|  | 5-60 minutes | -0.14 | 0.16 |  |  |
|  | Day 4-5 | 0.00 | 0.14 |  |  |
| Rainfall | | 0.08 | 0.15 | 0.26 | 0.61 |
| Group Size | | -0.11 | 0.11 | 0.91 | 0.34 |
| Breeding status | Babysitting | 0.00 | 0.00 | 4.77 | 0.31 |
|  | Escorting | 0.06 | 0.26 |  |  |
|  | Non-breeding | -0.47 | 0.37 |  |  |
|  | Oestrus | -0.29 | 0.49 |  |  |
|  | Pregnant | -0.08 | 0.40 |  |  |

Table S16 – Model predicting the frequency of grooming interactions during an observation, based on data from the day of the presentation. Model was fitted using a Poisson error structure and a log link function, with observation times as an offset term and trial ID as random intercepts, and an observation-level random intercept (GLMM, N = 44 trials in 5 groups). Significant terms are given in bold.

| Parameter | | Estimate | Standard Error | Χ^2^ | P |
| --- | --- | --- | --- | --- | --- |
| Intercept | | -2.47 | 0.54 |  |  |
| Treatment type:Stimulus type:Time point | |  |  | -0.44 | 1.00 |
| Stimulus type | Live intruders | 0.00 | 0.00 | 0.73 | 0.39 |
|  | Scents and calls | -0.16 | 0.19 |  |  |
| Treatment type:  Time point | Intrusion:5-60 minutes | -0.97 | 0.38 | **6.19** | **0.01** |
| Rainfall | | -0.02 | 0.14 | 0.03 | 0.87 |
| Group Size | | 0.07 | 0.02 | **11.01** | **<0.001** |
| Breeding status | Babysitting | 0.00 | 0.00 | 8.51 | 0.07 |
|  | Escorting | 0.18 | 0.31 |  |  |
|  | Non-breeding | -0.14 | 0.32 |  |  |
|  | Oestrus | 0.04 | 0.55 |  |  |
|  | Pregnant | 0.80 | 0.33 |  |  |

Table S17 – Model predicting the frequency of aggression interactions during an observation, based on the data from the day of the presentation. Model was fitted using a Poisson error structure and a log link function, with observation times as an offset term and trial ID as random intercepts, and an observation-level random intercept (GLMM, N = 44 trials in 5 groups). Significant terms are given in bold.

| Parameter | | Estimate | Standard Error | Χ^2^ | P |
| --- | --- | --- | --- | --- | --- |
| Intercept | | -4.38 | 0.70 |  |  |
| Treatment type:Stimulus type:Time point | |  |  | 0.69 | 0.41 |
| Treatment type:Time point | |  |  | 0.01 | 0.94 |
| Stimulus type | Live intruders | 0.00 | 0.00 | 0.37 | 0.54 |
|  | Scents and calls | 0.14 | 0.23 |  |  |
| Treatment type | Control | 0.00 | 0.00 | **13.32** | **<0.001** |
|  | Intrusion | 1.72 | 0.45 |  |  |
| Time point | 0-5 minutes | 0.00 | 0.00 | **11.67** | **<0.001** |
|  | 5-60 minutes | 0.83 | 0.26 |  |  |
| Rainfall | | -0.34 | 0.18 | 3.37 | 0.07 |
| Group Size | | 0.06 | 0.02 | **6.46** | **0.01** |
| Breeding status | Babysitting | 0.00 | 0.00 | 9.01 | 0.06 |
|  | Escorting | 0.63 | 0.39 |  |  |
|  | Non-breeding | 0.37 | 0.41 |  |  |
|  | Oestrus | -1.58 | 0.81 |  |  |
|  | Pregnant | 0.35 | 0.44 |  |  |

Table S18 – Model predicting the frequency of collective scent marking during an observation, based on data from the day of the presentation. Model was fitted using a Poisson error structure and a log link function, with observation times as an offset term and trial ID as random intercepts, and an observation-level random intercept (GLMM, N = 44 trials in 5 groups). Significant terms are given in bold.

| Parameter | | Estimate | Standard Error | Χ^2^ | P |
| --- | --- | --- | --- | --- | --- |
| Intercept | | -2.40 | 0.46 |  |  |
| Treatment type:Stimulus type:Time point | |  |  | 0.35 | 0.56 |
| Treatment type:Time point | |  |  | 0.04 | 0.85 |
| Stimulus type | Live intruders | 0.00 | 0.00 | 0.35 | 0.55 |
|  | Scents and calls | -0.09 | 0.15 |  |  |
| Treatment type | Control | 0.00 | 0.00 | 0.73 | 0.39 |
|  | Intrusion | 0.25 | 0.29 |  |  |
| Time point | 0-5 minutes | 0.00 | 0.00 | 0.43 | 0.51 |
|  | 5-60 minutes | -0.14 | 0.21 |  |  |
| Rainfall | | -0.02 | 0.12 | 0.04 | 0.85 |
| Group Size | | -0.02 | 0.02 | 2.16 | 0.14 |
| Breeding status | Babysitting | 0.00 | 0.00 | 0.75 | 0.95 |
|  | Escorting | -0.16 | 0.28 |  |  |
|  | Non-breeding | -0.05 | 0.28 |  |  |
|  | Oestrus | 0.18 | 0.42 |  |  |
|  | Pregnant | -0.08 | 0.29 |  |  |

Table S19 – Model predicting the frequency of collective alarm calling during an observation, based on data from the day of the presentation. Model was fitted using a Poisson error structure and a log link function, with observation times as an offset term and trial ID as random intercepts, and an observation-level random intercept (GLMM, N = 44 trials in 5 groups). Significant terms are given in bold.

| Parameter | | Estimate | Standard Error | Χ^2^ | P |
| --- | --- | --- | --- | --- | --- |
| Intercept | | -2.21 | 0.49 |  |  |
| Treatment type:Stimulus type:Time point | |  |  | 0.03 | 0.85 |
| Treatment type:Time point | |  |  | 0.03 | 0.86 |
| Stimulus type | Live intruders | 0.00 | 0.00 | 0.17 | 0.68 |
|  | Scents and calls | 0.07 | 0.32 |  |  |
| Treatment type | Control | 0.00 | 0.00 | **4.71** | **0.03** |
|  | Intrusion | 0.71 | 0.32 |  |  |
| Time point | 0-5 minutes | 0.00 | 0.00 | 0.80 | 0.37 |
|  | 5-60 minutes | -0.20 | 0.22 |  |  |
| Rainfall | | -0.13 | 0.13 | 0.96 | 0.33 |
| Group Size | | -0.04 | 0.02 | **4.60** | **0.03** |
| Breeding status | Babysitting | 0.00 | 0.00 | 4.16 | 0.38 |
|  | Escorting | -0.37 | 0.30 |  |  |
|  | Non-breeding | -0.35 | 0.30 |  |  |
|  | Oestrus | -0.05 | 0.44 |  |  |
|  | Pregnant | -0.57 | 0.31 |  |  |


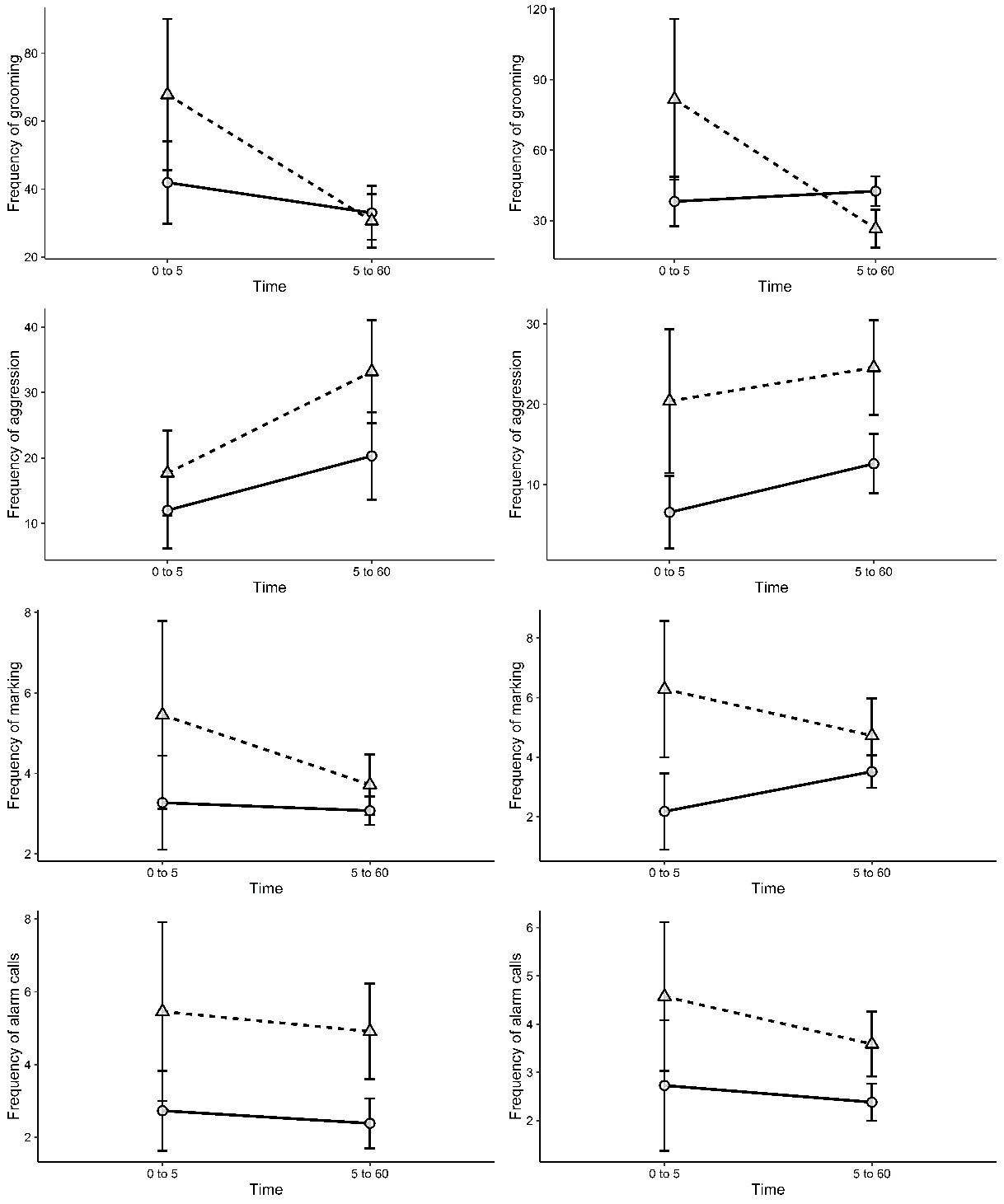


Figure S1 – Means and standard errors across time points after scents and calls (left) and live intruders (right) presentations. Open triangles and dashed lines show intrusion presentations, open circles and solid lines show stimulus control presentations.
